# Plant architecture without multicellularity: quandaries over patterning and the soma-germline divide in siphonous algae

**DOI:** 10.3389/fpls.2015.00287

**Published:** 2015-04-24

**Authors:** Viktoriya Coneva, Daniel H. Chitwood

**Affiliations:** Donald Danforth Plant Science Center, St. Louis, MO, USA

**Keywords:** morphology, multicellularity, coenocyte, green algae, transcript accumulation, patterning, small RNAs

## Abstract

Multicellularity has independently evolved numerous times throughout the major lineages of life. Often, multicellularity can enable complex, macroscopic organismal architectures but it is not required for the elaboration of morphology. Several alternative cellular strategies have arisen as solutions permitting exquisite forms. The green algae class Ulvophyceae, for example, contains truly multicellular organisms, as well as macroscopic siphonous cells harboring one or multiple nuclei, and siphonocladous species, which are multinucleate and multicellular. These diverse cellular organizations raise a number of questions about the evolutionary and molecular mechanisms underlying complex organismal morphology in the green plants. Importantly, how does morphological patterning arise in giant coenocytes, and do nuclei, analogous to cells in multicellular organisms, take on distinct somatic and germline identities? Here, we comparatively explore examples of patterning and differentiation in diverse coenocytic and single-cell organisms and discuss possible mechanisms of development and nuclear differentiation in the siphonous algae.

## Introduction

**FIGURE 1:**
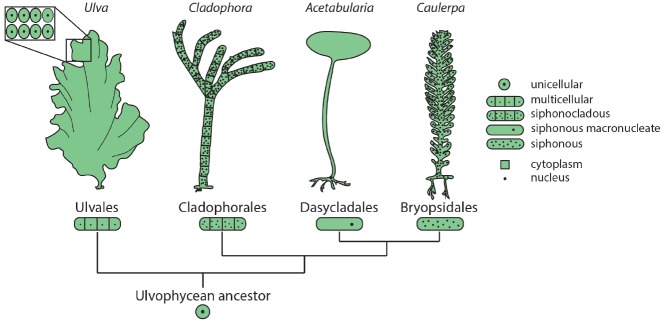
**Diverse morphologies and cellular organization in the green algae.** Orders within class Ulvophyceae contain examples of multicellular organisms (*Ulva*), siphonocladous species with multinucleate, multicellular organization (*Cladophora*), giant uninucleate cells (*Acetabularia*), and multinucleate siphonous algae (*Caulerpa*). The relationships among Ulvophycean classes (not drawn to scale) are based on the molecular phylogeny of Cocquyt et al. (2010).

Among the green plants (Viridiplantae), green algae (Chlorophyta) show remarkable diversity in morphology and cellular organization. Members of class Ulvophyceae include truly multicellular organisms, such as sea lettuce (*Ulva*) and various terrestrial green algae (e.g., *Trentepohlia*), macroscopic uninucleate cells, such as *Acetabularia*, giant siphonous coenocytes harboring multiple nuclei, such as *Caulerpa*, and siphonocladous species such as *Cladophora*, which are multicellular and multinucleate (Figure 1). Phylogenetic reconstruction suggests that the common ancestor of these species was a unicellular, uninucleate organism (Cocquyt et al., 2010), demonstrating that in the green plants macroscopic growth forms independently arose from diverse cellular strategies.

Indeed, although land plants (Embryophyta) are truly multicellular organisms, they possess siphonous-like properties at the cellular level (Kaplan and Hagemann, 1991; Niklas et al., 2013). In contrast to animal cell division, cytokinesis in land plants involves phragmoplast formation, which leaves cytoplasmic connections between daughter cells. This symplastic connectivity is further maintained via the formation of plasmodesmata. Beyond cytoplasmic connectivity, several lines of evidence suggest that land plant morphology is specified at an organismal level rather than through emergent properties of cells, lessening the contribution of multicellularity to plant architecture compared to animal morphology. For example, irradiation studies and clonal analyses have demonstrated that organ initiation and elaboration are largely independent of cell lineage patterns (Haber, 1962; Foard et al., 1965; Foard, 1971; Poethig, 1987). Furthermore, the phenomenon of compensation ensures that drastic changes in the cell cycle have remarkably little effect on overall organ shape in land plants (Tsukaya, 2006). Finally, morphological convergence over large evolutionary distances, between land plants, which exhibit true three-dimensional parenchymatous tissue patterning, red algae (Rhodophyta), which grow by two-dimensional filament elaboration, and unicellular siphonous growth in the green algae (Chlorophyta) highlights the relative independence of organismal morphology from cell division patterns (Kaplan and Hagemann, 1991). Thus, siphonous algae allow an opportunity to explore basic mechanisms enabling the elaboration of complex morphologies independent of histogenesis, and provide insights into the evolution of diverse plant architectures.

Two green algae, *Acetabularia acetabulum* (Dasycladales), which is a uninucleate single-celled organism throughout most of its lifecycle, and *Caulerpa taxifolia* (Bryopsidales), a multinucleate siphonous coenocyte, show striking patterns of local transcript accumulation superimposed on morphological structures convergent with the roots, stems, and leaves of land plants (Vogel et al., 2002; Ranjan et al., 2015). How siphonous algae achieve the feat of establishing compartmentalized transcript accumulation without cellularization of the cytoplasmic space remains largely unexplored. Although transcript partitioning is likely necessary for the elaboration of complex morphology in *Caulerpa* the fact that this organism consists of a single cell poses fundamental questions: (1) How widespread is mRNA movement throughout the *Caulerpa* coenocyte?; (2) Are nuclei in the shared cytoplasm functionally—or even genetically—equivalent?; and (3) How is the division between germline and soma achieved within a single cell, if at all? Here, we explore evolutionary solutions to these challenges in diverse organisms and outline hypotheses to examine the mechanisms underlying the complex architectures found in the siphonous algae.

## Patterning in a Single Cell: Morphology via mRNA Movement

Morphogenesis in multicellular organisms involves the establishment and maintenance of spatiotemporal transcript accumulation patterns. In the absence of cellular compartmentalization, localized gene expression or regulated transcript movement must act to create the mRNA patterns observed in *Caulerpa* and *Acetabularia* (Vogel et al., 2002; Ranjan et al., 2015), while processes such as tethering and regulation of protein translation may be in place to maintain them.

Interestingly, the *C. taxifolia* transcript accumulation profile shows a marked enrichment of RNA polymerase II transcripts in the holdfast, a pseudo-organ analogous to roots in land plants (Ranjan et al., 2015). As RNA polymerase II is a necessary component of the transcription machinery, a tempting hypothesis is that transcription either exclusively or preferentially occurs in holdfast nuclei, while transcripts observed throughout the rest of the cell have moved. Examples of regulated intracellular mRNA movement are well documented and often mechanistically dependent on the cytoskeleton. Actin filaments are employed for unidirectional mRNA translocation, such as polar localization of transcripts regulating asymmetric mating type switching in *Saccharomyces cerevisiae* (Paquin et al., 2007) and hyphal tip growth of filamentous ascomycete fungi, while microtubule-associated transcript movement enables long-distance bi-directional localization in the coenocyte of the basidiomycete fungus *Ustilago maydis* (Zarnack and Feldbrügge, 2010). Classical *Acetabularia* grafting experiments by Hämmerling (1953) provided the first clear demonstration that the single basal nucleus, through its implied molecular action at a distance, specifies cap morphology in this unicellular macroalga. Recent studies have demonstrated that actin-dependent mRNA movement is at least one component of this long-distance patterning signal during vegetative growth (Mine et al., 2001). Similarly, localized transcription and subsequent transcript movement may generate spatio-temporal transcript partitioning in the *Caulerpa* coenocyte (Ranjan et al., 2015). Nuclear run-on assays and targeted disruption of actin polymerization can determine if indeed transcription is restricted solely to the holdfast. In addition to cytoskeleton-associated transcript movement, the prevalence of cytoplasmic streaming in *Caulerpa* suggests that bulk cytoplasmic flow may aid the transport of patterning machinery (Sabnis and Jacobs, 1967; Herth et al., 1972; Kuroda and Manabe, 1983; Menzel, 1987). Live imaging approaches to simultaneously track microinjected labeled mRNAs and to visualize distinct cellular components can address the extent of transcript movement in *Caulerpa* and the contributions of cytoplasmic streaming and the cytoskeleton to this process.

## Many Nuclei, One Cytoplasm: Heterogeneity and Local Subfunctionalization

Local gene expression differences within a single multinucleate cell may arise due to the existence of genetically distinct nuclear populations harboring varying *cis*-regulatory alleles. Some filamentous fungi, for example, actively maintain a diverse nuclear population within a single cell (Roper et al., 2011). An important implication of nuclear heterogeneity is that it necessitates coordination of nuclear activities to decrease intra-organismal genetic conflict (Michod and Roze, 2001; Roper et al., 2011; Niklas, 2014). During vegetative growth of filamentous ascomycetes, for example, heterokaryosis through hyphal fusion between distinct genotypes is regulated by an elaborate somatic incompatibility checking system (Roper et al., 2011). In discussing the possibility of nuclear heterogeneity in *Caulerpa*, the fact that these species are capable of regenerating the entire coenocyte from any vegetative fragment emerges as an important consideration. Moreover, in *C. taxifolia* sexual reproduction is limited, and there are reports that the invasive strain may only be male (Phillips, 2009). Since vegetative propagation predominates, nuclear inheritance is mainly cytoplasmic and the accumulation of spontaneous mutations defining multiple intracellular nuclear lineages within a single *Caulerpa* coenocyte is likely. This would create a high genetic burden and conflict between distinct nuclear lineages, and blur the distinction between individual and the population. As we discuss subsequently, a soma germline divide may maintain transgenerational genomic integrity. Additionally, mechanisms to rein in genomic diversity within a single coenocyte may also exist. Indeed, genome-wide comparisons of individual nuclei in the asexually reproducing endomycorrhizal fungus *Rhizophagus irregularis* show remarkable inter-nuclear homogeneity (Lin et al., 2014). Cycles of genome rearrangements or parasexual nuclear recombination and gene conversion may contribute to diversification and homogenization in the absence of sex in these fungi and other asexual organisms (Lin et al., 2014; Seidl and Thomma, 2014). Currently, there is little evidence of the precise nuclear make-up of a single *Caulerpa* species (see Varela-Álvarez et al., 2012, for a comparison of genome size and ploidy across species). Thus, the extent of intracellular nuclear genetic diversity in *C. taxifolia* needs to be resolved in order to address outstanding questions, including its impact on organismal morphology, reproduction, and population genetics. A consensus *Caulerpa* genome and genomic resequencing of single nuclei will be instrumental in understanding nuclear population dynamics in the *Caulerpa* coenocyte.

If, indeed, nuclear genetic diversity is limited within the *Caulerpa* coenocyte, it is interesting to speculate that groups of nuclei may somehow become locally committed to different expression patterns. Nuclear subfunctionalization is supported by the observation of “nucleo-cytoplasmic domains”—regularly spaced nuclei hypothesized to represent local zones of influence—in many syncytia and coenocytes (Goff and Coleman, 1987; McNaughton and Goff, 1990; Bruusgaard et al., 2003; Anderson et al., 2013). The demonstration of exclusive associations between individual nuclei and the plasma membrane during syncytial stages of *Drosophila melanogaster* embryogenesis is further evidence that the establishment of functional nuclear domains may be a means of spatial subfunctionalization in a shared cytoplasm (Mavrakis et al., 2009). Moreover, the formation and maintenance of the morphogenic bicoid gradient in *Drosophila* (Driever and Nüsslein-Volhard, 1988) is intricately intertwined with local nuclear dynamics (Gregor et al., 2007). These examples illustrate that, analogous to cells in multicellular organisms, the delineation of nuclear zones of influence may be a hallmark of coenocytic and syncytial growth. An intriguing implication of this observation is that in the absence of physical cell boundaries, nucleo-cytoplasmic domains, instead of cells, may form an elemental, subcellular functional unit of eukaryotic life (Baluška et al., 2004), consistent with the organismal theory proposed by Kaplan and Hagemann (1991).

## Reconciling Nuclear Activity and Potential: A Possible Role for Small RNAs

As discussed in the previous section, the prevalence of vegetative propagation in *C. taxifolia* may necessitate mechanisms to rein in the effects of spontaneous mutations. Moreover, regeneration implies that fragments derived from any portion of the coenocyte can give rise to the entire organism. Thus, similar to the ability of differentiated cells of land plants to revert to a totipotent state, mechanisms to preserve nuclear potential may be in place in *Caulerpa*. However, recent evidence that transcripts with nuclear-related functions (such as transcription, DNA replication, chromatin, and RNAi) are highly enriched in the stolon and holdfast regions (Ranjan et al., 2015) suggests that nuclear populations in *Caulerpa* may be functionally distinct. To reconcile these observations, it is tantalizing to speculate of a nuclear division of labor into transcriptionally active and inert nuclei, analogous to the specification of somatic and germline cellular fates.

The setting aside of specialized germ cells, which act as propagules of a “true” copy of an organism’s genome, provides a buffer from the variance to which somatic genomes are subject. The use of such a divide is amplified with increasing organismal size and life span, which correlate with increased propensity for the accumulation of deleterious somatic variants (Michod and Roze, 2001; Umen, 2014). In *C. taxifolia*, which as discussed, may be a long-lived clonal lineage, the need for a mechanism to reduce the transgenerational effects of deleterious somatic variants is particularly acute given that genetically distinct nuclear populations may be present in a single cell.

Germline fidelity is intimately connected with small RNAs and their mobility, leading to speculation about the significance of the observed restriction of transcripts related to RNAi biogenesis to *Caulerpa* stolons (Ranjan et al., 2015). A conceptual basis for how small RNA-mediated nuclear germline identity may be accomplished within a shared cytoplasm is provided by the angiosperm microgametophyte. During pollen formation in *Arabidopsis thaliana*, a vegetative and a generative nucleus share a common cytoplasm in the microspore. The generative nucleus gives rise to sperm nuclei, which ultimately contribute their genomes to the next generation. Transposable elements are activated in the vegetative nucleus allowing for the generation of small interfering RNAs (siRNAs). siRNAs accumulate in sperm nuclei and direct transposon silencing ensuring transgenerational genomic fidelity (Slotkin et al., 2009). Although mechanistically distinct, a small RNA-dependent phenomenon is also involved in inter-nuclear communication during conjugation in ciliated protozoans, which exhibit nuclear dimorphism with a transcriptionally inert germline micronucleus and an active somatic macronucleus. Ciliates undergo conjugative sexual reproduction during which micronuclei recombine and are transmitted to the next generation, while somatic macronuclei are degraded. A special feature of macronuclei, which likely contributes to their role as transcriptionally active entities, is that site-specific DNA rearrangements result in the removal of ∼15% of the micronucleus genome from which the zygotic macronucleus is derived. Interestingly, the site-specificity of these macronuclear genome rearrangements is guided by a specific class of small RNAs, termed scnRNAs (Mochizuki et al., 2002; Duharcourt et al., 2009; Fang et al., 2012) illustrating another context in which small RNAs act to coordinate inter-nuclear division of labor and to preserve germline integrity.

**FIGURE 2:**
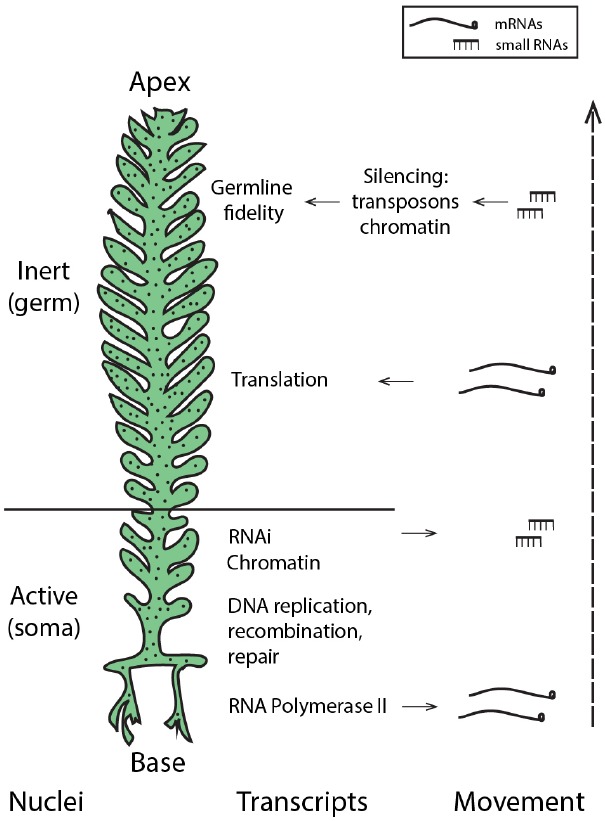
**Hypothetical model: localized transcription and regulated movement of mRNAs and small RNAs may pattern the *Caulerpa* coenocyte.** Transcriptionally active nuclei in the stolon and frond base produce transcripts and small RNAs. mRNA movement, coupled with local tethering and translation outside the region of transcription, establish spatio-temporal patterning. Small RNAs move from the stolon apically to induce silencing in the frond pinnules, creating germline-like, transcriptionally inert nuclei at the site of gametogenesis. “Nuclei” denotes hypothesized nuclear states; localized accumulation of transcript classes (as described in Ranjan et al., 2015) is shown by “Transcripts” and “Movement” shows the acropetal transport of mRNAs and small RNAs.

The presence of core machinery for small RNA-mediated silencing in the Ulvophycean lineage (Cerutti et al., 2011) supports the possibility that small RNA-directed pathways may be used by siphonous green algae such as *Caulerpa*. Moreover, miRNAs in *Chlamydomonas reinhardtii* peak in abundance during gametogenesis (Zhao et al., 2007), suggesting a role for small RNA pathways as not only regulators of development and morphology, but perhaps guardians of the germline in this Chlorophyte. Thus, generating a small RNA atlas of the *Caulerpa taxifolia* coenocyte will be instrumental in evaluating the possibility that small RNAs may pattern nuclei with somatic and germline characteristics and maintain genomic fidelity. While enrichment of RNAi-related transcripts in the *Caulerpa* stolon suggests that small RNA production may be localized to this pseudo-organ (Ranjan et al., 2015), the extent and mechanisms of small RNA mobility and its local accumulation in the shared cytoplasm remain open questions. Active nuclei in the stolon, acting analogously to somatic cells, might produce small RNAs that travel to silence nuclei in the pinnules, where gametogenesis is localized (Figure 2).

## Conclusion and Perspectives

Siphonous green algae exhibit remarkable morphological complexity, convergent with the architecture of land plants. The elaboration of form in a single cell highlights the necessity to address the mechanisms by which morphology arises in a supra-cellular, organismal context. At the very least, morphology requires the establishment and maintenance of spatiotemporal gene expression patterns. In multicellular organisms, developmentally regulated variation in gene expression defines cellular, tissue, and organ levels of organization. Evidence suggests that transcript partitioning contributes to morphological complexity in coenocytic organisms as well. How such patterns are generated within a shared cytoplasm remains speculative. Additionally, the implications of cytoplasmic inheritance of the nuclear genome on intracellular genetic diversity and conflict, organismal morphology, and the maintenance of genomic fidelity are all open questions. Precedent from studies in diverse multinucleate organisms helps to outline a conceptual basis of development and inheritance in siphonous algae. Small RNA movement may locally specify nuclear activity, while transcript movement and tethering may contribute to differential gene expression and morphological patterning. The generation of genomic resources, a small RNA expression atlas, as well as development of live imaging and cytoplasmic manipulation techniques in giant coenocytes such as *Caulerpa* will provide a strong foundation to address these hypotheses. Ultimately, understanding the mechanisms by which complex morphology arises in the absence of multicellularity has fundamental implications for our understanding of the evolution of plant architecture.

### Conflict of Interest Statement

The authors declare that the research was conducted in the absence of any commercial or financial relationships that could be construed as a potential conflict of interest.
